# The pectinolytic activity of *Burkholderia cepacia* and its application in the bioscouring of cotton knit fabric

**DOI:** 10.1186/s43141-023-00596-5

**Published:** 2023-11-23

**Authors:** Sabrina Samad Shoily, Kaniz Fatema, Rasheda Begum Dina, Anik Biswas, Papia Haque, Mohammed Mizanur Rahman, Md. Zulhash Uddin, Abu Ashfaqur Sajib

**Affiliations:** 1 Department of Genetic Engineering and Biotechnology, University of Dhaka, Dhaka, Bangladesh; 2 Department of Wet Process Engineering, Bangladesh University of Textiles, Dhaka, Bangladesh; 3 Department of Applied Chemistry and Chemical Engineering, University of Dhaka, Dhaka, Bangladesh; 4 Institute of Leather Engineering and Technology, Dhaka, Bangladesh

**Keywords:** *Burkholderia cepacia*, Pectinase, Pectinolytic enzymes, Bioscouring, Pectin

## Abstract

**Background:**

Enzymatic catalysis in different industrial applications is often preferred over chemical methods due to various advantages, such as higher specificity, greater efficiency, and less environmental footprint. Pectinases are a group of enzymes that catalyze the degradation of pectic compounds, the key components of plant middle lamella and the primary cell wall. Pectinases have found applications in multiple industrial processes, including cotton bioscouring, fruit juice extraction and its clarification, plant fiber degumming, paper making, plant biomass liquefaction, and saccharification, among others. The purpose of this study was to taxonomically characterize a bacterial species exhibiting pectinolytic activities and assess its pectinolytic activity qualitatively and quantitatively, as well as test its bioscouring potential.

**Results:**

Here, we report that *Burkholderia cepacia*, a previously unknown species with pectinolytic activity, exerts such activity comparable to commercially used pectinase enzymes in the textile industry, but requires less temperature for activity.

**Conclusion:**

Quantitative evaluation of enzyme activity indicates the potential of the bacterial species for use in the bioscouring of cotton knit fabric.

**Supplementary Information:**

The online version contains supplementary material available at 10.1186/s43141-023-00596-5.

## Background

Pectinases are a unique group of enzymes that catalyze the degradation of pectic compounds, such as pectins, propectins, pectinic acid, and pectic acid [1, 2]. Pectic substances, which consist of long galacturonic acid pillar chains, are essential constituents of the middle lamella and primary cell wall of plants [2, 3]. Pectinases are naturally involved in the metabolic activities of plants, fungi, and bacteria [2]. In plants, they play a role in multiple processes, including cell wall metabolism and extension, cell growth and senescence, ripening of fruits, and tissue softening at the time of maturation [3]. They act by catalyzing the breakdown of glycosidic bonds of the long chains of galacturonic acid residues in pectic substances and converting the polygalacturonic acid into monogalacturonic acid [2].

Enzymatic catalysis is preferred over chemical methods in several industries due to various advantages, such as high specificity, high catalytic efficiency, less aggressiveness, and adjustable activity [3]. Pectinases have drawn attention from researchers around the world because of their application as a biological catalyst in multiple industrial processes, including fruit juice extraction and its clarification, cotton scouring, plant fiber degumming, plant biomass liquefaction and saccharification, wastewater treatment, tea and coffee fermentations, paper making, and vegetable oil extraction [1, 4]. This enzyme is primarily derived from plants and microorganisms [2]. Microbial pectinases comprise 25% of the worldwide market for food and industrial enzymes, and their value is consistently increasing [3]. Nevertheless, researchers continue to encounter challenges in the areas of identifying unique microbial pectinases, elucidating their mechanisms of action, and increasing production capacity. Bacterial pectinases were shown to have activity in industrial settings [2], and many bacteria still remain uncharted for their pectinolytic activity. The discovery of pectinolytic activity in bacteria and the associated genes may improve industrial processes to be more sustainable.

Raw cotton consists of on average 92% cellulose, along with a complex mixture of non-cellulosic natural impurities, such as fats, waxes, pectins, and proteins [5]. These impurities impart hydrophobic nature and interfere with aqueous chemical processes on cotton, including dyeing and finishing [5, 6]. In order to generate fibers that are highly wettable and suitable for uniform finishing and dyeing, hydrophobic impurities are eliminated through the alkaline scouring process. This process entails subjecting the cotton to simmering with hot aqueous sodium hydroxide [5]. Nevertheless, this procedure not only eliminates non-cellulosic impurities but also undermines the structure of the fabric through its attack on the cellulose [7].

Bioscouring is an energy-efficient and ecologically sustainable technique that eliminates non-cellulosic impurities from fiber by increasing the hydrophilic nature of the fiber surface through the action of particular enzymes [5, 8]. This method circumvents the negative impacts of cellulose depolymerization and fiber strength degradation, as well as ecosystem contamination that is linked to the application of abrasive chemicals in conventional alkaline scouring. The most complex noncellulosic constituent in the fiber’s primary wall is pectin, which can be degraded and solubilized using pectinase enzymes [5]. Pectinases can be divided into two main groups: acidic and alkaline pectinases. Acidic pectinases are produced mainly by fungi, and these enzymes can be used for the clarification of fruit juices, whereas alkaline pectinases are produced primarily by alkalophilic bacteria [9] and are effective in bioscouring of natural cellulosic fibers such as cotton, hemp, linen, and blends [7, 8]. As a result, it is critical to identify pectinase-producing bacterial strains and assessing the potential of pectinase derived from these bacteria in bioscouring and enhancing fabric wettability.

Bioconversion of lignocellulosic biomass, which is a promising renewable feedstock for biofuel production, can be achieved through a series of processes: physicochemical pretreatment and lignin removal to expose cellulose and hemicellulose, hydrolysis of carbohydrate polymers to produce free sugars, and fermentation of free sugar to produce bioethanol [10, 11]. The enzymatic hydrolysis of cellulose is influenced by its accessibility [10]. Pectin degradation methods must be optimized in order to efficiently produce biofuels from raw biomass because pectin can influence the accessibility of other cell wall components to enzymatic degradation due to the cellulose-hemicellulose network in the cell wall being embedded in a pectin matrix [12]. Pectinase degrades pectic substances present in cell walls, lowering medium viscosity and softening tissues [13]. A previous study demonstrated that the removal of pectin from fiber hemp by pectinase treatment increased cell wall surface, improving the accessibility of cellulose to degradative enzymes and enhancing conversion to free sugars [14]. As a result, screening bacterial strains capable of producing pectinase may be useful in biotechnological approaches for using lignocellulosic biomass for biofuel generation.

The purpose of this study was to taxonomically identify a bacterial strain capable of producing pectinase and assess its pectinolytic activity qualitatively and quantitatively, as well as test its bioscouring potential.

## Materials and methods

### Isolation of pure cultures of bacteria

The microbial cultures used in this study had been obtained from a spurious growth in selectable minimal medium plates (NaCl 6.0 g/l, (NH_4_)_2_SO_4_ 1 g/l, KH_2_PO_4_ 0.5 g/l, K_2_HPO_4_ 0.5 g/l, MgSO_4_.7H_2_O 0.2 g/l, CaCl_2_.2H_2_O 0.1 g/l) using 1.2% agar supplemented with pectin (0.5% w/v) as the sole carbon source, which was originally prepared for the selection of a specific recombinant bacterium. Pure cultures were obtained using the Luria–Bertani (LB) agar medium (1.5% (w/v) agar and 2% (w/v) LB) through multiple subcultures.

### Screening of pectinase-producing bacteria and qualitative assessment of pectinase activity

Bacteria from pure culture plates were streaked on a minimal agar medium containing pectin (0.5%) as the sole carbon source. In each of these pectin-minimal agar media plates, the non-pectinolytic *Escherichia coli* was inoculated as a control. Following incubation of these streaked plates at 37 °C for 48 h, pectin degradation was determined by flooding the plates with freshly prepared 0.1% (w/v) Congo Red solution in water, followed by de-staining with 1 M NaCl solution. A clear zone around the growth indicated the ability to break down pectin. To further assess pectinase activity, this strain was streaked on a pectin-LB-agar medium (2% (w/v) LB, 1.2% (w/v) agar, and 0.5% (w/v) pectin) [15]. Here, as well, two non-pectinolytic *E. coli* bacterial strains were used as negative controls. One of these was a natural strain of *E. coli*, and the other was a transformed *E. coli* harboring a pGLO plasmid.

### Evaluation of the growth characteristics

A single colony of the pectin-degrading isolate was inoculated into 2% LB (w/v) broth in wells of a new 96-well plate. The plate was then incubated in a shaking incubator at 37 °C with orbital shaking at 140 rpm for 26 h. The optical density (OD) at 600 nm was measured at intermittent times over the period using a microplate reader (Gentaur/GDMS, Belgium), and a growth curve was generated by plotting the ODs at different time points [16].

### Assessment of pectin-degrading activity and thermal stability of crude pectinase enzyme

Following bacterial growth in minimal broth medium with 0.5% pectin at 37 °C in a rotary shaker at 140 rpm, cells were precipitated by centrifugation at 4000 × g for 10 min at 4℃ in 50 ml tubes, washed with freshly prepared cold phosphate-buffered saline (PBS) (003002, Invitrogen) and then resuspended in PBS. Cells were ruptured by sonication on ice and cell-free crude extract was collected following centrifugation at 4000 × g for 10 min at 4℃ in 50 ml tubes. The total protein concentration was determined by measuring absorbance at 280 nm using a UV/Vis Spectrophotometer (OPTIMA SP-3000nano, Japan). Pectinase activity in crude extract was evaluated following the Nelson-Somogyi method [17, 18]. The pectin degrading activity was evaluated at four different temperatures (30, 40, 50, and 60 °C), each with three replicates. For each replicate, crude enzyme (1 mL) was mixed with 0.2 mL of 0.5% pectin in Eppendorf tubes and then incubated in heat blocks for 2 h. With 1 mL of this incubated solution, 2 mL of Somogyi copper reagent [19] was added, and the tubes with the mixture were set in boiling water for 10 min. The tubes were cooled to room temperature and 1 mL of the Nelson arsenomolybdate reagent [19] was added. The Somogyi Copper reagent was prepared with the absorbance of molybdenum blue and was measured at 520 nm using the UV/Vis spectrophotometer. A standard curve for measuring the released reducing sugar was generated using known concentrations of d-glucose. The Somogyi Copper reagent was formulated using 18% (w/v) Na_2_SO_4_ (Wako Pure Chemical Industries Osaka, Japan), 1.2% potassium sodium tartrate (w/v) (Wako Pure Chemical Industries Osaka, Japan), 2.4% Na_2_CO_3_ (w/v) (Merck Darmstadt, Germany), 0.4% CuSO_4_.5H_2_O (w/v) (Merck Darmstadt, Germany), and 1.6% NaHCO_3_ (w/v) (Merck Bombay, India). The Nelson arsenomolybdate reagent was prepared using 5% (NH_4_)_6_Mo_7_O_24_.4H_2_O (w/v) (Carl Roth, Karlsruhe, Germany), 0.6% AsHNa_2_O_4_.7H_2_O (BDH, Germany), and 4.2% Benzoic acid (Merck, Darmstadt, Germany).

### Molecular identification and characterization of the screened isolate

The pectinase-producing bacteria were cultured in LB (2% (w/v)) broth at 37 °C for 16 h in a shaking incubator at 140 rpm. The culture was centrifuged at 16,000 × g for 10 min in a 1.5-ml tube, the supernatant was removed, and the pellet was washed with 500 µl of PBS, followed by centrifugation at 16,000 × g for 10 min and removal of the supernatant. The pellet was then mixed with 150 μl of 10% (w/v) Chelex^®^-100 (C7901, Sigma) in ultrapure water, vortexed vigorously, and incubated at 95 °C for 10 min with intermittent vortexing. The solution was centrifuged at 16,000 × g for 10 min, and the supernatant with the genomic DNA was collected for amplifying the 16S ribosomal RNA gene (rDNA) [20].

16S rDNA gene sequence was amplified in a reaction total reaction volume of 25 μl containing 1.5 μl of the DNA sample), 2.5 μl of 10 × PCR buffer (EP0702, Thermo Fisher Scientific), 1.0 μl of dNTP mix (10 mM), 0.5 μl of each forward (16S_357F: CTCCTA CGGGAGGCAGCAG) and reverse (16S_1100R: AGGGT TGCGCTCGTTG) primers (10 μM), 0.2 μl of Taq DNA polymerase (EP0702, Thermo Fisher Scientific), and nuclease-free water using a thermal cycler following an initial denaturation step at 94℃ for 3 min, then 30 cycles—each with denaturation at 94℃ for 30 s, annealing at 61℃ for 1 min, and elongation at 72℃ for 1 min., followed by the final extension at 72℃ for 5 min. The amplified products were resolved in 1.2% (w/v) agarose gels using 1 × Tris Acetate EDTA (TAE) buffer along with a DNA size marker (300003, GeneON). Amplified DNA sequences were visualized in a gel documentation system following electrophoresis in a 1.2% (w/v) agarose gels [21]. PCR products were purified using the FavorPrep™ GEL/PCR Purification Kit (FAGCK 001, Favorgen Biotech Corp.) following the manufacturer’s protocol, and purified PCR products were sequenced following the Sanger sequencing with a commercial service. To identify the nearest neighbors of the pectinase-producing isolate, the resulting DNA sequence was subjected to the NCBI Basic Local Alignment Search Tool (BLAST) [22]. The BLAST search was restricted to 16S rRNA (Bacteria and Archaea). Additionally, models (XM/XP) and uncultured/environmental samples were also filtered out. In the MEGA11 software [23], this sequence was aligned with the top 50 high-similarity sequences obtained from the BLAST search using the ClustalW program [24], and a phylogenetic tree was constructed by the neighbor-joining (NJ) method with 1000 bootstrap.

### Enzyme activity tests on fabric

Single jersey cotton knit fabric samples of 155 GSM (gram per square meter) were collected from Microfibre Group, Bangladesh. The pre-treatment auxiliary detergent KS-10 was collected from Tubingen Chemicals (BD) Ltd., Bangladesh. Reactive dye (Rb. Red 3BX) and other chemicals that were used in the dyeing solution (46 g/l Glauber salt, 18 g/l soda ash, 10 g/l leveling agent, 10 g/l sequestering agent, and 20 g/l anti-creasing agent) were collected from Orient-Chem Ltd., Bangladesh. Pectinase enzyme for use as control was collected from Tubingen Chemicals (BD) Ltd., Bangladesh. The commercial enzyme is usually used in the textile industry for fabric bioscouring as an amount of 1 g/l with 0.2 g/l detergent in a pretreatment bath (pH 7.9) for 60 min at 60 °C, and then, a hot wash is done with 0.5 g/l detergent in wash bath for 20 min at 90 °C. The pH value was 6.9 when the fabric was treated within a bath containing the 1 g/l crude extract with 0.2 g/l detergent for 60 min at 37 °C, and the same hot washing process was carried on the crude extract treated fabric. In enzymatic treatment fabric weight was 20 gm, and the material-to-liquor ratio was 1:10. Both of the enzyme-treated fabric samples were dyed according to industrial recipe with 2% reactive dye at 60 °C for 90 min. Here, the fabric weight was 5 gm and the material-to-liquor ratio was maintained at 1:10. As per the instruction of the dyeing recipe, the dye was taken based on the weight of the fabric and all chemicals with auxiliaries were measured for 50 ml. Excluding soda ash, other chemicals, auxiliaries, dyes, and fabric were put on the dye pot with the adjustment of water for 50 ml and set in the dyeing machine at 60 °C. Soda ash was not added at this stage to prevent alkaline hydrolysis of reactive dyes. Thirty minutes later, soda ash was added to the dye bath and run for an additional 60 min. Dyed fabrics were washed following sequential cold wash, acid wash (50% acetic acid, 1 g/l), first hot water wash, soap wash, second hot water wash, and third hot water wash to achieve good colorfastness of the dyed sample. Cold wash was done for 7 min at room temperature, and each hot wash was run for 7 min at 95 °C. ECO dyer (ECO-18, Xiamen Rapid Co., Ltd. China) was used for the enzymatic pre-treatment and dyeing processes.

#### Wettability test

The absorbency of the enzyme-treated cotton fabric was evaluated using the vertical wicking test (AATCC 197, 2022 Edition, 2022), and the level of absorbency of the enzyme-treated cotton fabric was determined by measuring the color absorption time and distance traveled by the liquid when a cut edge of the fabric is submerged into 1% dye solution.

#### Measurement of color difference

Using the principle of the International Commission on Illumination (usually abbreviated CIE for its French name, Commission internationale de l'éclairage), a spectrophotometer (datacolor 850) was utilized to determine the color difference (variations in shades) between collected and prepared pectinase enzyme-treated samples [25]. If the color difference (DE) is equal or less than 1, the shade is counted acceptable [26].

#### Fourier transform infrared spectroscopy (FT-IR) analysis

FT-IR spectrometer (ABB MB3000) was used to analyze the fabric samples. The untreated and enzyme-treated cotton samples were folded which was then scanned individually and recorded separately. The scanned data was transferred to Origin software (OriginLab Corp.) and plotted transmittance percentage against wave number (cm^−1^) values.

### Statistical analysis

All the experiments were performed using at least three replicates. All statistical analyses were performed using Microsoft Excel and GraphPad Prism (version 6) software. The data are presented as mean ± standard deviation.

## Results and discussion

### Bacterial isolate with pectin degrading abilities

**Fig. 1:**
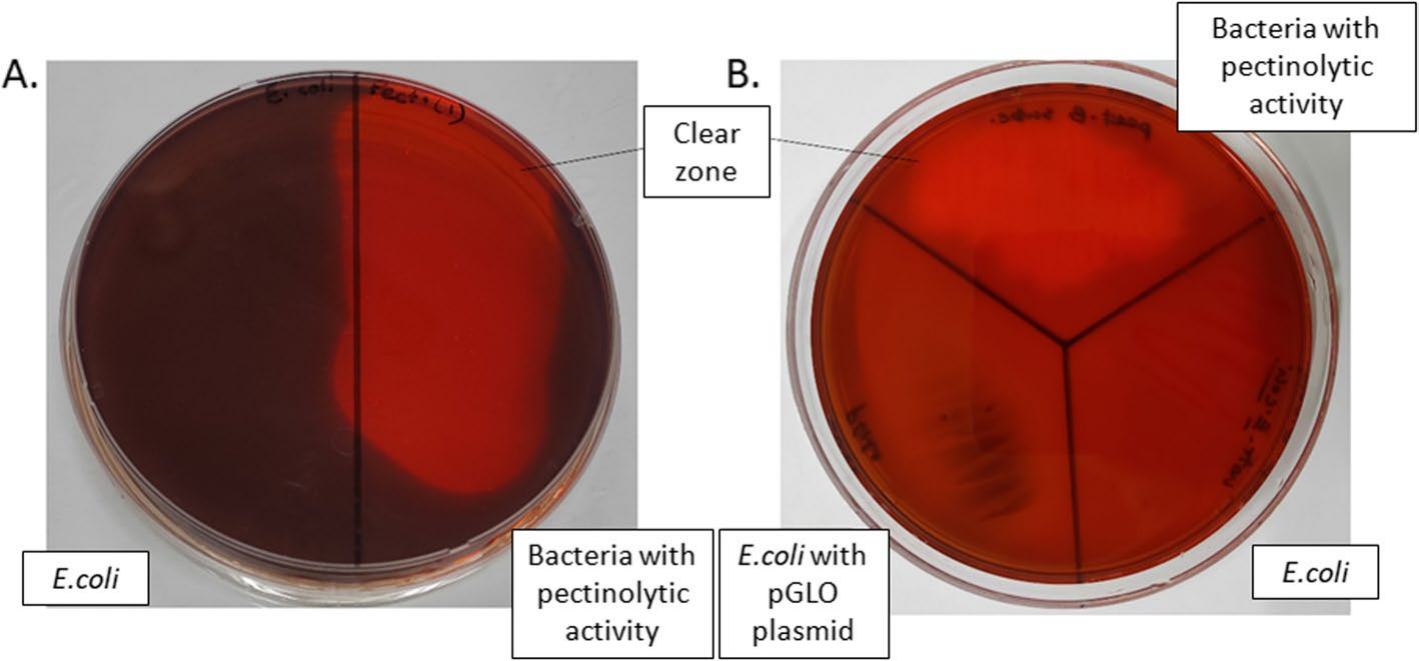
Screening of pectinolytic activity using the Congo red assay. A clear zone was observed around the colonies of pectinase-producing bacteria in (**A**) minimal agar plates with pectin as the sole carbon source and (**B**) pectin-Luria–Bertani (LB) agar media. No clear zone was observed for *E. coli* as well as transformed *E. coli* harboring pGLO plasmid

**Fig. 2:**
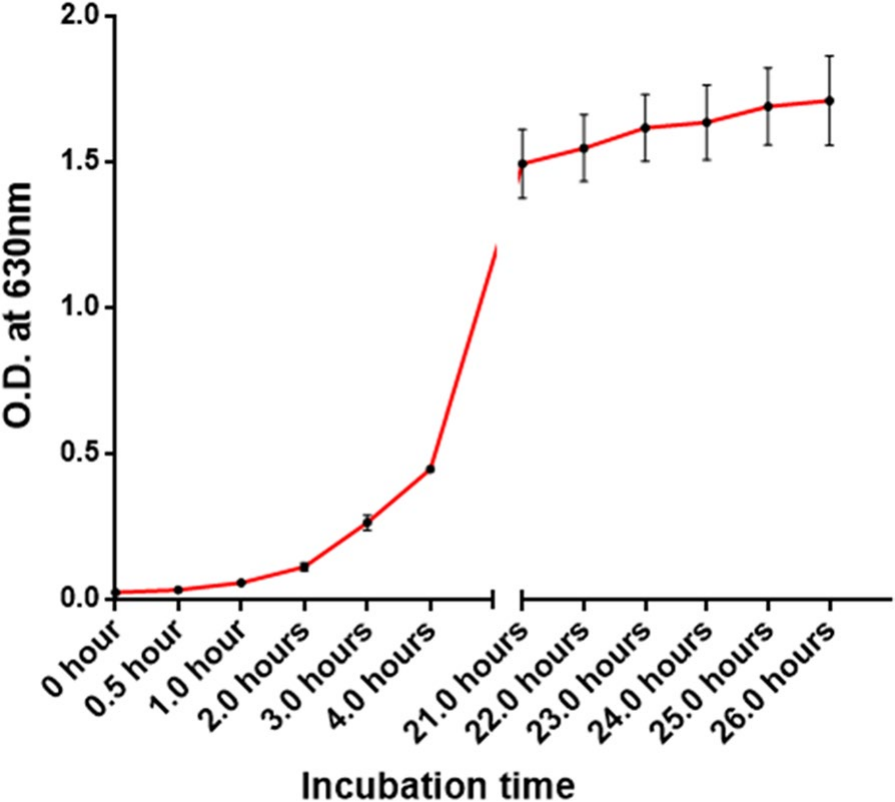
Growth curve of the pectinolytic bacterial isolate

The bacterial isolate selected on a pectin-minimal medium showed pectinolytic activity in the Congo red assay (Fig. 1a). The clear zone formed around the bacterial growth demonstrated the isolate’s pectin degradation capabilities. In the region where the control *E. coli* strains grew, no such zone was observed. This activity was corroborated further by detecting a comparable clear zone in the Congo red-washed pectin-LB-agar plate with the screened bacteria (Fig. 1b). The pectinase-producing isolate exhibited typical growth characteristics of bacteria (Fig. 2).

### Activity of the crude enzyme extract at different temperatures

**Fig. 3:**
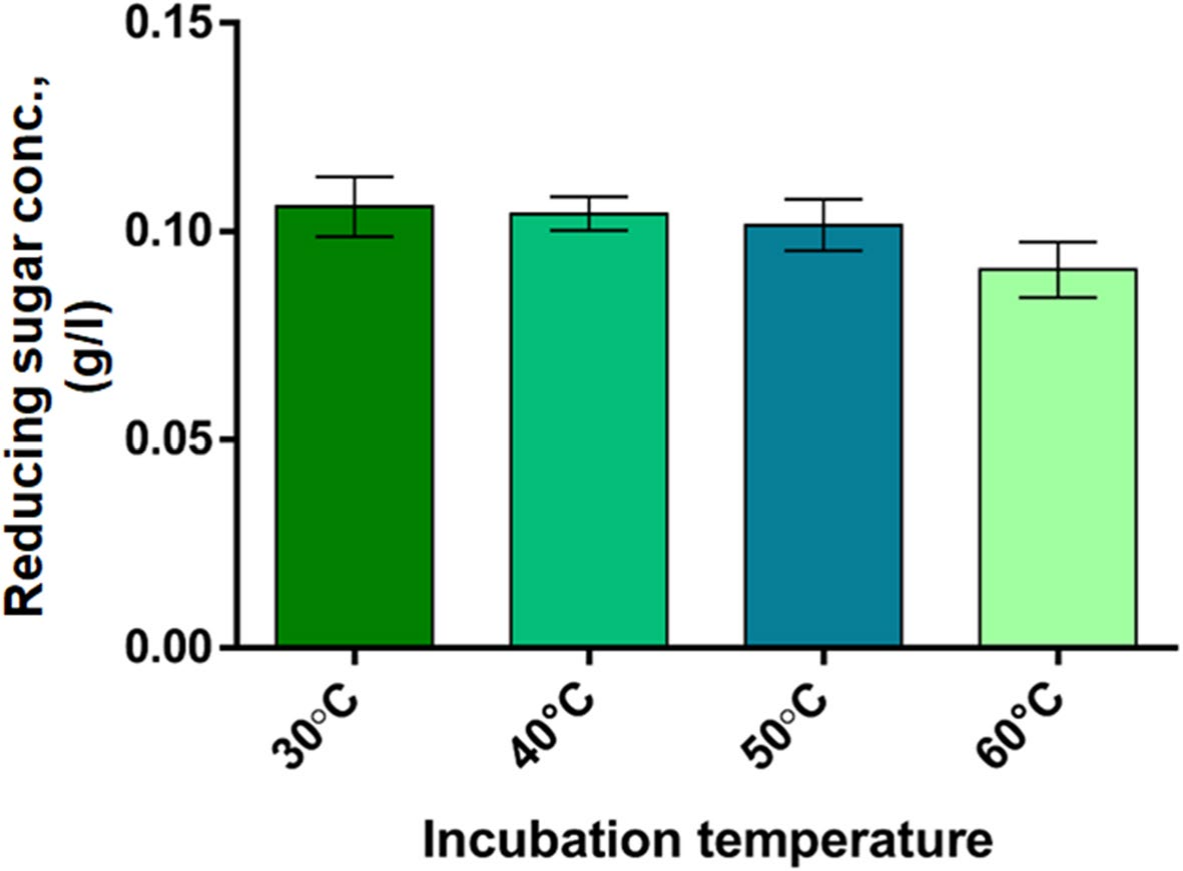
Pectinolytic activity of cell-free crude extract

d-galacturonate is released from pectin during the degradation of pectin. d-galacturonate is a reducing sugar and its release from pectin due to pectinolytic activity was assessed at various temperatures. In Fig. 3, the amount of monomers of pectin (Galacturonate) released was compared to a reducing sugar standard curve. The highest yields were obtained at temperatures between 30 and 40℃, with mean amounts of liberated reducing sugar exceeding 100 mg per liter.

### Molecular identity of pectinase-producing bacteria

16S rRNA sequence similarity search using BLAST revealed the highest similarity (~ 99.9% identity) of the bacterial sequence with *Burkholderia cepacia.*

Multiple sequence alignments with 50 of the best hits from this search produced a neighbor-joining tree shown in Supplementary Figure 1. It showed the highest closeness with the species of *B. ubonensis*, *B. dolosa*, *B. latens*, *B. multivorans*, *B. cenocepacia*, *B. vietnamiensis*, *B. metalica*, *B. cepacia*, and *B. terrtorri* (Supplementary Figure 1).

### Activity of the cell-free crude extract on cotton fabric

**Fig. 4:**
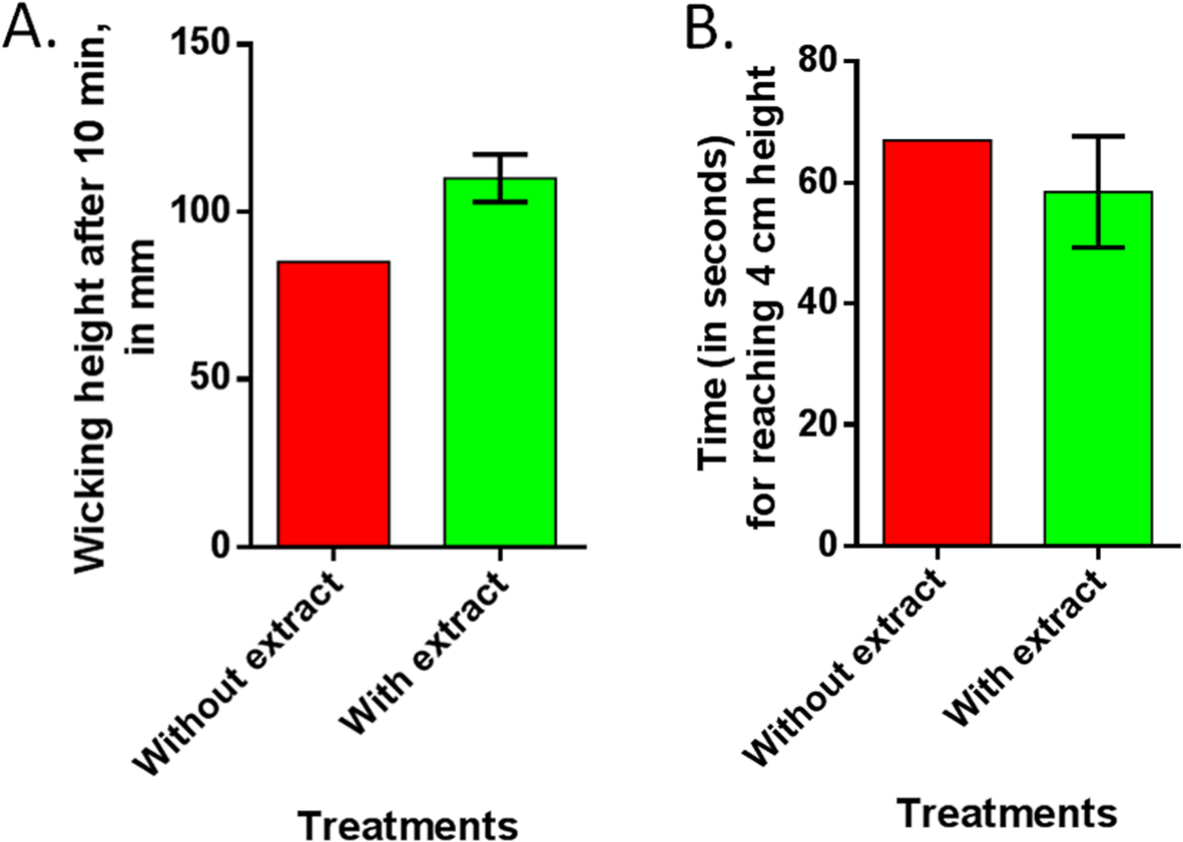
Wicking test. **A **Wicking height for fabric samples treated without and with enzyme. **B** The required time to reach 4 cm at the wicking test for untreated and treated fabrics

A wicking test was conducted to observe the wicking rate in the untreated and enzyme-treated fabric (Fig. 4). The average wicking rate is encouraging because of an increased wicking height in the case of enzyme-treated fabric than untreated fabric at the same parameters and washing conditions. Surfactant adds to the process of scouring through the removal of waxes and fats at high temperatures, while the enzyme facilitates the removal of pectic substances [27]. In the wicking test, water traveled the largest distance in the cellular extract-treated fabric compared to the controls (Fig. 4). In line with this experiment, the water took the least time (in seconds) to travel 4 cm in height in the cellular extract-treated fabric.

### The color difference of control and pectinase enzyme-treated samples

Color difference CMC DE (color difference developed by Color Measurement Committee) values between control and cell extract-treated samples for 2% depth of color are given in Table 1. Following the CIE Lab principle, the L*, a*, b*, c*, and h^o^ values were measured by the difference between the values of dyed samples from commercially available enzyme and cellular extract-treated fabric, which is then converted into the CMC DE value. The acceptable limit of the CMC DE value is ≤ 1. The color difference values of the control and the cell extract-treated dyed sample were within the acceptable range.

**Table 1:** The color difference between control and pectinolytic enzyme-treated dye-stained samples

Sample	CIE L values	CIE a values	CIE b values	CIE c values	CIE h values	CIE DE values	CMC DE values
Control enzyme, 1 g/l	47.27	50.21	6.31	50.61	7.17	1.07	0.51
Cell extract 1 g/l	47.56	50.92	7.06	51.40	7.89		

The light transmittance of each sample was measured. In this assay, particular attention needs to be given to the aliphatic C–H stretch indicator at wavelengths of 2923 cm^−1^ and 2885 cm^−1^, hydroxyl group’s 3288 cm^−1^, and ester carbonyl’s 1732 cm^−1^. These groups are present in pectin, as well as the wax and the cuticle layer of the cotton fibers. Cellulose is surrounded by a cuticle which is composed of mainly wax and cutin; pectin acts as reinforcement among the constituents and bridges the cuticle with the cell walls [28]. Effective pectic compounds’ degradation can lead to a decrease in these substances and an increase in the transmittance values at those wavelengths.

**Fig. 5:**
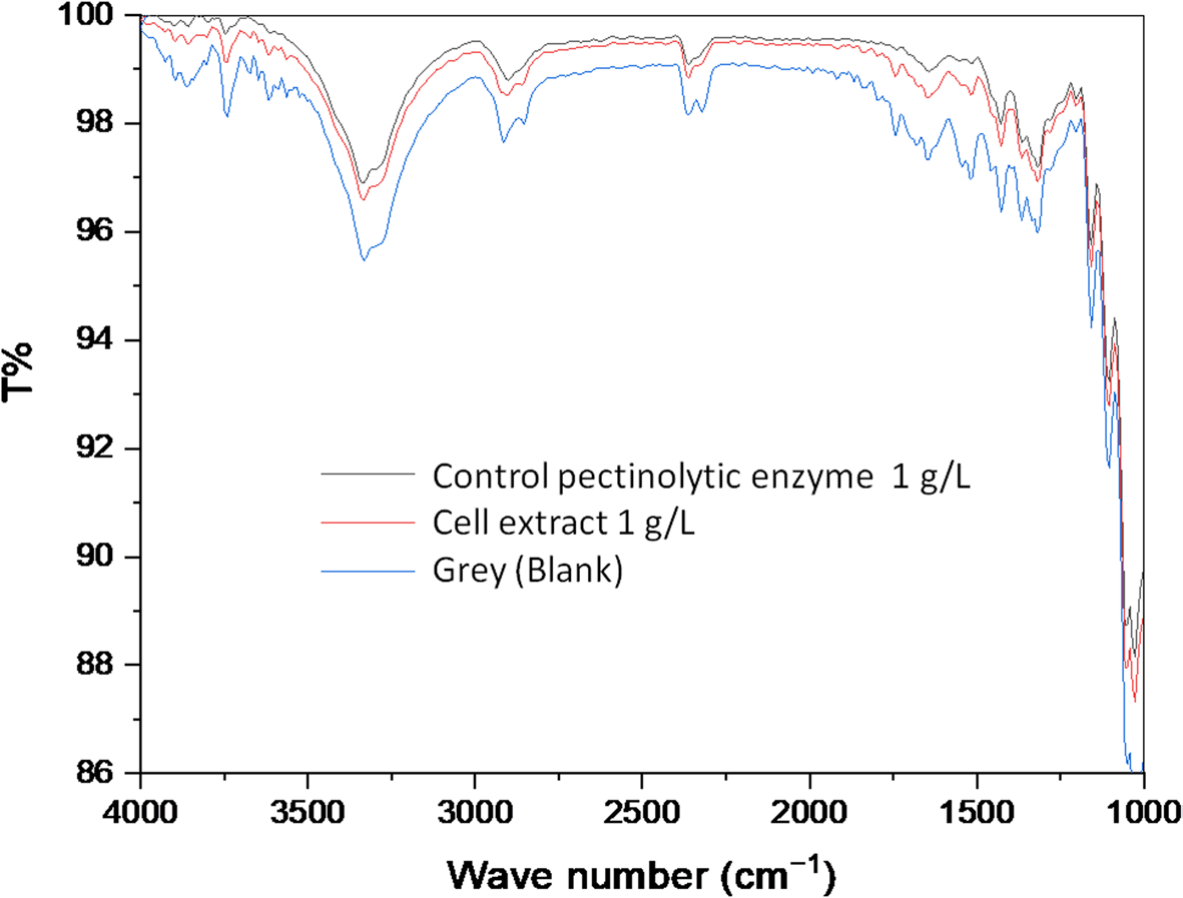
Transmittance percentage against wave number values. The line identified as “gray” represents the untreated control. BS enzyme refers to the commercial used enzyme

The graph from Fig. 5 shows the weaker intensity of the characteristic bands of aliphatic CH stretch, hydroxyl, and ester carbonyl wax and the cuticle layer of cotton. The mono and tri-esoteric components like wax, fat, and oil were removed with the detergent action. The weakened and moved-out bands in the region of 1740 ~ 1200 cm^−1^ refer to the partial removal of pectin [29]. The intensity of the peak is changed significantly in the mentioned region from gray, and the bands are almost identical to the commercial enzyme.

## Discussion

The textile industry heavily relies on pectinase for scouring the cotton fabrics. Novel enzymes are needed with ready availability, increased efficacy for gentler fabric processing, and reduced process cost. We have attempted to study such a pectinase enzyme that came from an organism spuriously growing on pectin-containing culture plates.

After preliminary analyses through repeated cultures, confirmation of the specific species was obtained through a local alignment search using the 16S rRNA gene sequence. 16S rRNA gene sequencing can be performed using a ubiquitous set of primers and local alignment is a powerful technique to mine the sequence database for similarity.

The result from a BLAST search indicated that the bacteria in question is most probably *Burkholderia cepacia*. This is an important finding since this species was not previously reported to harbor pectin degradation activity. Multiple sequence alignment through neighbor-joining and subsequent bootstrapping is an established vigorous procedure to remove alignment errors, and the results of which show a similar outcome.

Its pectinase enzyme might have potential characteristics useful for textile and other industries. To investigate these abilities, several tests were undertaken. The crude extracts were used to find the optimal temperature for activity and were found to be between 30 and 40 °C, much lower than the ones currently used in the textile processing. As a result, lower temperatures can be used for bioscouring, significantly reducing the cost for heating purposes to reach optimum fabric treatment.

The practical application of the crude extract from this organism is promising because of an increased wicking height in the conventional wicking test to measure the wettability of fabrics. This increase in wicking height can be attributed to the breakdown of pectic substances by enzymatic treatment and removal by hot wash. The greater the wicking height, the more the wettability of the fiber. As shown in Fig. 4, treatment with cellular extract increased the wettability of fabric, which was comparable to the performance of a commercially available pectinase enzyme used in the textile industry (data not shown).

The results indicate that during the treatment with pectinase, the enzyme assisted in removing pectin and also allowed the rapid access of detergent into the fiber in the next step of hot wash at 90℃. Surfactant is a necessary component in enzyme-based bioscouring and has an impact on the removal of waxes and fats at high temperatures, after the enzyme facilitates the removal of the pectic substances.

The dye retention efficacy of the final fabric was also measured using the established methods of the Color Measurement Committee and showed acceptable results for quality products. Fourier transform infrared spectroscopy is another method that can show the abundance of chemical bonds in the molecular level, and the results from our analyses is proof of the fact that the relative abundance of bond associated with pectic substances is much lower in the treated samples.

The discovery of the pectinolytic ability in this bacterial species can indeed lead to a better textile processing method, requiring less temperature for bioscouring of the raw cotton and producing better quality fabrics with dye retainability. Further research is needed to know the activity of the enzyme in alkaline condition that occurs during cotton processing. Long-term research methodologies can include fabric strength measurements and long-time wear-and-tear studies.

## Conclusion

Bacteria are the major source of alkaline pectinases, which are used in textile processing and bio-scouring of cotton fibers, degumming and retting of fiber crops, pretreatment of pectic wastewaters from fruit juice industries, coffee and tea fermentations, paper making, and enzyme based oil extraction. Most of the fungal pectinases are acidic and unsuitable for use in industrial processes requiring neutral to alkaline pH conditions. Additionally, bacterial strains are preferred over fungal strains for industrially relevant enzyme production because of the ease of the scale-up process, strain improvement, and application of other modern approaches to increase production yield. In this study, we have identified a previously unreported bacterial species capable of producing pectin-degrading enzymes. Quantitative evaluation of enzyme activity indicated the potential of the strain to be used for an industry-level production of pectinase enzyme. Additionally, the application of the enzyme on raw cotton indicated that this new enzyme shows comparable activity to commercially available ones, but requires less temperature for activity.

### Supplementary Information

**Additional file 1: Figure S1.** Neighbor-joining (NJ) tree based on the 16S rRNA gene sequence of the selected pectinolytic bacteria. The numbers on the branches of the NJ tree represent bootstrap support values. NCBI GenBank accession numbers of the individual sequences are written in front of each species name.

## Data Availability

All data generated or analyzed during this study are included in this published article.

## Abbreviations

pGLO: A recombinant plasmid that harbours the green fluorescent protein
OD: Optical density
RPM: Rotation per minute
FDR: False discovery rate
LB medium: Luria-Bertani medium
w/v: Weight/volume
