# Supplementary figures and images for: The pectinolytic activity of Burkholderia cepacia and its application in the bioscouring of cotton knit fabric
Source: J Genet Eng Biotechnol. 2023 Nov 23;21:136. doi: 10.1186/s43141-023-00596-5 (PMC10667187; doi:10.1186/s43141-023-00596-5)

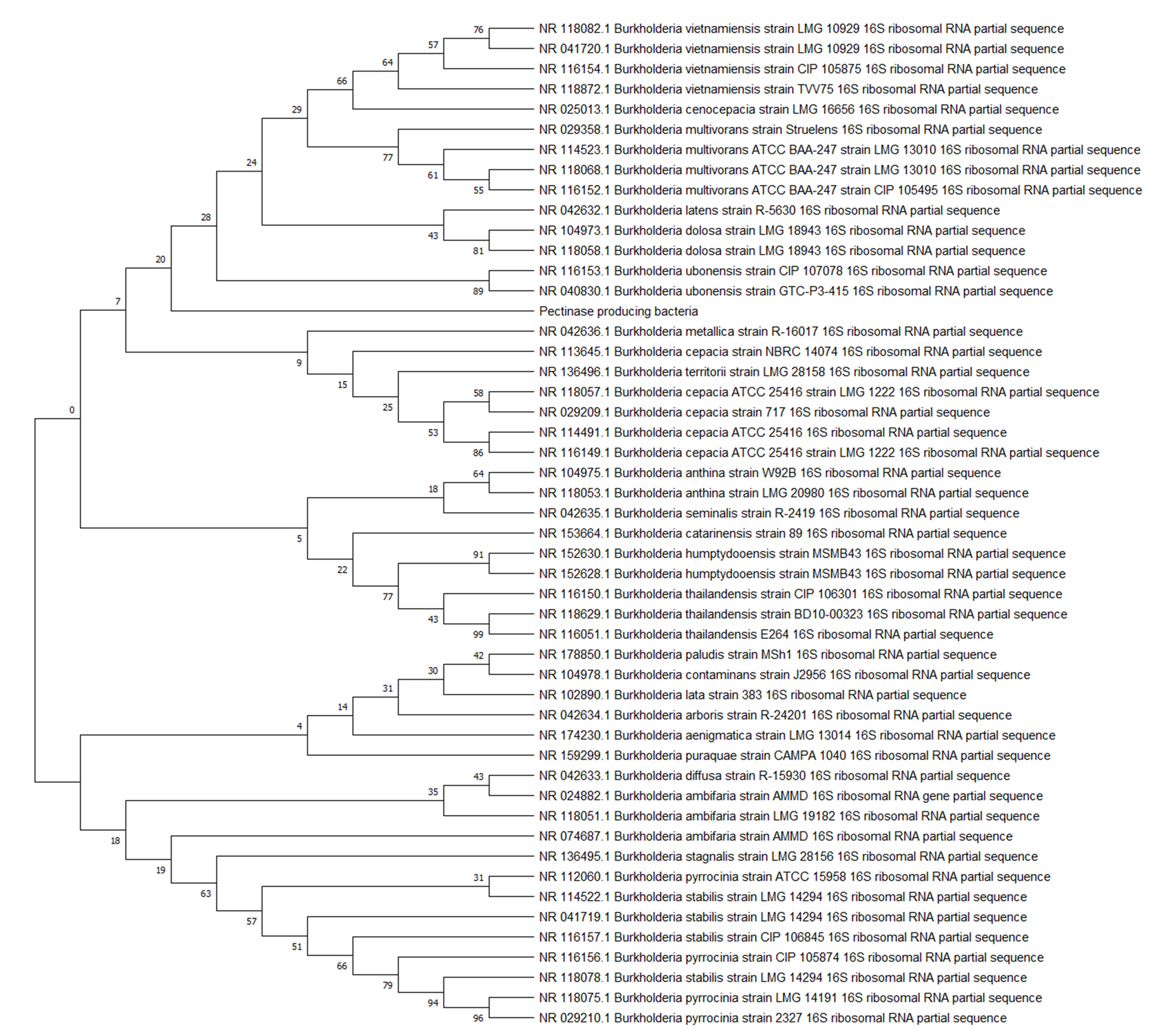

Supplement: Supplementary file 1 — Additional file 1: Figure S1. Neighbor-joining (NJ) tree based on the 16S rRNA gene sequence of the selected pectinolytic bacteria. The numbers on the branches of the NJ tree represent bootstrap support values. NCBI GenBank accession numbers of the individual sequences are written in front of each species name. [file 43141_2023_596_MOESM1_ESM.tif]
